# External Microplastic Filters for Washing Machines: Advances, Technical Challenges, and Biofouling Mitigation

**DOI:** 10.3390/membranes16070238

**Published:** 2026-07-13

**Authors:** Hyeri Song, Yeo Min Kim, Da Hyun Yoo, Sun Ae Kim, Chanhyuk Park

**Affiliations:** 1 Department of Environmental Science and Engineering, Ewha Womans University, Seoul 03760, Republic of Korea; 2 Department of Food Science and Biotechnology, Ewha Womans University, Seoul 03760, Republic of Korea

**Keywords:** capture, filter, laundry wastewater, microplastic, nanoplastic, washing machine

## Abstract

Laundry-derived microplastic fibers are major source of environmental microplastic pollution. Because conventional wastewater treatment processes cannot completely remove fibrous microplastics, washing machine-mounted filters have been developed as a promising source-control approach for the reduction in microfiber emissions. This review thus summarizes recent advances in external filtration systems and membrane-based technologies for laundry wastewater treatment. The characteristics of laundry wastewater, microfiber release behavior, and recent regulatory trends are discussed together with the performance of commercially available filtration systems. The applicability of advanced membrane materials, including ceramic membranes, for high-efficiency microfiber separation is also highlighted. Particular attention is given to the microbial contamination and biofouling of microplastic filters, which can affect their filtration efficiency, operational stability, and household hygiene. This review also discusses the potential reuse of captured microplastics for membrane fabrication as a sustainable pathway for waste valorization and circular resource utilization. Finally, current limitations and future perspectives for the development of efficient, hygienic, and sustainable laundry microplastic filtration technologies are discussed.

## 1. Introduction

Microplastics released from laundry wastewater are a significant source of environmental pollution, particularly in urban aquatic systems [1], with the synthetic microfibers generated during domestic washing processes becoming one of the dominant contributors to microplastic contamination [2]. In particular, a single laundry cycle can release millions of microfibers into wastewater streams [3], and textile washing is estimated to account for approximately 35% of primary microplastics entering the oceans [4]. These fibers, which are typically smaller than 5 mm, originate primarily from synthetic textiles such as polyester, nylon, and acrylic, and their release has increased with the growing global demand for synthetic clothing [5,6].

A major concern associated with microplastic fibers is their persistence and resulting environmental impact [7]. Due to their resistance to biodegradation, microfibers accumulate in aquatic environments and pose risks to both ecosystems and human health [4,8]. Microplastics can act as carriers for hazardous pollutants, microorganisms, and algae, facilitating their transport and potentially forming toxic aggregates through adsorption [8,9,10,11]. Aquatic organisms can also ingest microfibers through filtration or misidentification as food, leading to physical damage such as intestinal blockage and inflammation [12,13,14,15]. Notably, microfibers have been reported to account for a significant proportion of the microplastics found in aquatic organisms, illustrating their ecological significance.

Once released, laundry-derived fibers are transported to wastewater treatment plants (WWTPs), where influent concentrations have been reported to range from 1 to 10,044 particles per liter, with maximum values reaching 31,400 particles per liter in some cases [16,17]. Despite the widespread use of conventional wastewater treatment processes, the removal of microplastic fibers remains incomplete [18]. Their length, flexibility, and low density allow them to remain suspended during primary treatment, while their irregular morphology reduces the efficiency of conventional filtration systems [4,5,19,20]. Although secondary biological processes can partially remove microplastics, their non-biodegradable nature limits the overall removal efficiency [14]. Advanced tertiary treatments, such as membrane bioreactors (MBRs) and rapid filtration, are capable of achieving high microplastic removal rates, but their high capital and operating costs have restricted their widespread implementation [21]. In addition, microplastics captured during treatment may accumulate in sewage sludge and be reintroduced into the environment if the sludge is used for land applications [22].

These limitations highlight the importance of source-control strategies that prevent the release of microplastics at the point of generation [23]. For this reason, external microplastic filters installed in washing machines have gained increasing attention as a practical and scalable solution. These systems capture microfibers directly from the laundry effluent before they enter wastewater streams [24]. Recent developments have focused on improving the capture efficiency of these filters by optimizing their pore size, filtration materials, and structural configurations. However, several technical challenges remain, including the pressure drop across the membrane, clogging, their relatively low durability, and high maintenance requirements [25]. In particular, biofouling, which is the accumulation of microorganisms and extracellular polymeric substances (EPSs) on the filter surface, is a serious but often overlooked factor influencing filter performance [26]. Because laundry wastewater contains a diverse microbial community and various forms of organic matter, microplastic filters can become sites for microbial attachment and biofilm formation [27]. Much of the current understanding of biofouling derives from membrane-based filtration systems and from the internal wetted components of household washing machines—including their built-in drain filters—where biofilm formation has been documented. Whether external microplastic filters are similarly susceptible has not yet been directly examined, and because laundry conditions such as surfactant load and fluctuating temperatures differ from those of conventional membrane systems, filter biofouling is treated here as an extrapolation that warrants dedicated investigation.

Several recent reviews have addressed laundry-derived microfiber pollution from complementary but distinct perspectives: Luzi et al. [28] critically examined the release of microfibers from textiles, emphasizing the washing-related factors that govern fiber shedding, the lack of standardized quantification protocols, and the legislative landscape, whereas Ramasamy et al. [29] systematically benchmarked commercial and laboratory-scale microfiber filters in terms of filtration efficiency, durability, disposal, and sustainability. Both works establish that point-of-use retention devices are promising yet limited by modest, variable capture efficiencies and considerable maintenance demands. In contrast, the present review approaches external washing-machine filters from a membrane-process and materials standpoint and extends the discussion to dimensions largely absent from prior reviews—namely, the application of established membrane materials such as ceramic microfiltration (MF) and ultrafiltration (UF) membranes as fouling-resistant, high-efficiency alternatives to conventional mesh filters, the microbial colonization and biofouling of microplastic filters and its implications for filtration performance and household hygiene, and the upcycling of captured microplastics into membrane materials—thereby positioning biofouling mitigation and resource valorization as central to the sustainable deployment of laundry microplastic filtration technologies.

This review provides a comprehensive overview of external microplastic filters for washing machines. It first introduces the characteristics of laundry wastewater and existing microplastic removal technologies, followed by a detailed analysis of recent advances in filter design, regulatory and policy trends, and key parameters governing the microplastic capture efficiency. Particular emphasis is placed on summarizing biofouling mechanisms and mitigation strategies designed to ensure sustainable and long-term filter performance. Finally, current technical limitations, future research directions, and the broader applicability of these technologies to other microplastic sources are discussed.

## 2. Characteristics and Treatment of Laundry Wastewater

### 2.1. Water Quality of Laundry Effluent

Laundry wastewater is a complex form of contaminated graywater or industrial effluent containing detergent residues, fabric-derived particles, organic matter, oils, grease, and trace metals [30,31], with its composition varying greatly depending on the operating conditions (e.g., laundry type, scale of operation, detergent formulation and dosage, washing temperature, and cycle frequency) [32,33]. In general, household laundry wastewater has lower pollutant loads and variability than that derived from commercial and industrial facilities such as hotels, hospitals, and large-scale laundries, which generally have higher concentrations of suspended solids (SSs), organic matter, surfactants, and dissolved ions [30]. Therefore, source-specific characterization is required to design effective treatment and reuse strategies and to meet discharge standards.

Previous studies have characterized laundry wastewater using key physicochemical parameters, including pH, biochemical oxygen demand (BOD), chemical oxygen demand (COD), total SS (TSS), total dissolved solids (TDS), electrical conductivity, turbidity, and surfactant concentration (Table 1). The pH of laundry wastewater is primarily governed by alkaline detergent builders such as sodium hydroxide, sodium silicate, and sodium carbonate [34]. Household laundry wastewater typically has a near-neutral pH (6–9), whereas commercial and industrial effluents have a wider range (pH 5–12), often with strong alkalinity [35,36,37,38]. In terms of organic load, household laundry wastewater generally has a BOD of 40–266 mg/L and a COD of 429–1755 mg/L [35,39,40,41], indicating a relatively high fraction of non-biodegradable organic matter compared to typical municipal wastewater. Commercial and industrial laundry wastewater tends to have higher organic loads, with a BOD of 190–542 mg/L and a COD of 323–1710 mg/L [35,37,42,43]. TSSs have also been reported to have a range of 141–469 mg/L in household wastewater and 60–1116 mg/L in commercial and industrial wastewater [35,36,39,44], which can impose a substantial burden on downstream treatment processes such as sedimentation, filtration, and membrane separation.

Laundry wastewater also has a high ionic strength due to its detergent components, as indicated by electrical conductivity and TDS measurements. Household laundry wastewater generally has a conductivity of 641–5760 µS/cm and TDS levels of 229–2000 mg/L, while the TDS levels of commercial and industrial wastewater can exceed 6000 mg/L in some cases [35,36,41,44,45,46]. Turbidity and color also vary widely depending on the source, with turbidity levels of 25–281 NTU found in household laundry wastewater, compared to 92–858 NTU for commercial and industrial wastewater [35,38,39,41]. Surfactants, particularly anionic surfactants such as linear alkylbenzene sulfonate (LAS), are key pollutants in laundry wastewater. Their concentrations range from approximately 16 mg/L in household laundry wastewater to 9–59 mg/L in commercial and industrial wastewater, with values as high as 164 mg/L reported depending on detergent usage and washing conditions [37,39,42,47]. These compounds can inhibit biological treatment processes, are toxic for aquatic environments, and can cause operational issues such as foaming [48,49].

Among the reported laundry wastewater characteristics, BOD, COD, TSS, and surfactant concentrations are the water quality parameters most relevant to external filter fouling. High BOD and COD indicate increased biodegradable and organic matter loading, which can facilitate microbial growth and biofilm development on filter surfaces [26]. Elevated TSS promotes the formation of cake layers that increase hydraulic resistance and reduce permeate flux [50,51]. In addition, residual surfactants can enhance the adsorption of organic matter and microbial adhesion, thereby accelerating fouling [27]. Therefore, these parameters should be considered in the design and operation of external washing-machine filters to achieve stable long-term filtration performance.

**Table 1:** Summary of the physicochemical characteristics of domestic, commercial, and industrial laundry wastewater worldwide.

Category	Country	pH	BOD (mg/L)	COD (mg/L)	TSS (mg/L)	Conductivity (μS/cm)	TDS (mg/L)	Turbidity (NTU)	Ref.
Domestic Laundry Wastewater	Oman	6.8–7.7	40–80	997–1204	260–525	1560–5760	428–2000	32.8–135	[35]
	India	8.5 ± 0.5	265.7 ± 142.9	1754.7 ± 1064.6	469.1 ± 289.4	-	1837.7 ± 552.6	281.8 ± 136.8	[39]
	Nigeria	8.8–9.0	144–211.2	428.6–531.3	228.9–460	-	229–461	25.3–39.4	[41]
	India	9.1 ± 1.4	186.5 ± 108.8	1545.8 ± 346.2	141.2 ± 35.6	641.6 ± 404.8	710.4 ± 125.2	108.6 ± 75.7	[36]
Commercial Laundry Wastewater	Oman	10.4–10.9	80–220	323–755	814–1116	4110–13,800	6380–8849	91.9–150	[35]
	Brazil	5.6 ± 0.9	-	1710 ± 968	80 ± 60	-	-	-	[37]
	Canada	10.4 ± 0.5	-	579.3 ± 30	60 ± 10	494 ± 20	-	110 ± 5	[44]
	USA	12.5 ± 0.5	-	1138 ± 58	359 ± 82	724 ± 123	357 ± 52	858 ± 111	[38]
	Poland	7.9–9.0	335–542	727–944	-	2009–5747	-	-	[43]
Industrial Laundry Wastewater	Poland	8.2	370–390	631–768	-	-	-	-	[47]
	France	6.5–9.0	445 ± 114	835 ± 176	<500	-	-	-	[52]
	-	7.5–8.5	190–210	686–1088	-	1800–2300	-	-	[42]

Values are presented as reported in the original sources, either as ranges (min–max) or as mean ± standard deviation.

### 2.2. Current Treatment Methods for Laundry Wastewater

The treatment of laundry wastewater depends strongly on its source and pollutant load. Household laundry wastewater is typically discharged as municipal sewage and treated at public WWTPs, whereas commercial and industrial effluents originating from laundries, hotels, hospitals, and textile facilities are classified as industrial wastewater and treated on-site or at centralized industrial treatment facilities [53]. Accordingly, treatment strategies vary in complexity and are selected based on influence characteristics, discharge regulations, reuse requirements, and economic feasibility [54,55]. Processes widely used for laundry wastewater treatment include coagulation–sedimentation, biological treatment, filtration, advanced oxidation, membrane separation, and electrochemical methods, which are often integrated into hybrid systems [55].

Conventional treatment typically proceeds through sequential stages of pretreatment, primary sedimentation, biological treatment, and secondary sedimentation, and emerging pretreatment technologies such as microbubble flotation have recently been investigated for the removal of microplastics and fine suspended solids [56,57]. The activated sludge process, which serves as the core technology in most wastewater treatment plants, achieves high removal efficiencies of approximately 90.8% for BOD and 95.5% for SS, and its microplastic removal efficiency has been reported to exceed 90% [58,59].

To meet stricter discharge standards or enable water reuse, tertiary treatment is implemented following biological treatment. Activated carbon adsorption effectively removes dissolved organic matter, surfactants, color, and odor [60,61], whereas advanced oxidation processes such as ozonation, UV/H_2_O_2_, and the Fenton reaction generate hydroxyl radicals that degrade refractory organic pollutants [62]. Industrial laundry wastewater, which is characterized by a high and highly variable pollutant load, often cannot meet discharge standards through a single process and thus requires multistage treatment integrating physicochemical and biological processes [31]. For instance, an extended aeration activated sludge (EAAS) process achieved removal efficiencies of approximately 90% for COD and 71% for surfactants, while a downstream UV/O_3_ oxidation unit further degraded the residual organic pollutants [42].

Despite significant advances in laundry wastewater treatment technologies, the complete removal of microfibers remains challenging. Owing to their elongated shape, flexibility, and low density, microfibers can remain suspended during treatment, allowing a fraction to escape with treated effluent or become concentrated in sewage sludge [18,22]. Consequently, relying solely on downstream wastewater treatment is insufficient to effectively prevent microfiber emissions into the environment. A complementary source-control strategy is therefore needed to capture microfibers at the point of generation before they enter the wastewater stream [23]. External microplastic filters installed on washing-machine drain lines provide a practical solution because they can be readily implemented at the household level without requiring additional centralized infrastructure. By capturing microfibers at their highest concentration, these filters can reduce the loading on wastewater treatment plants while limiting microfiber release into receiving aquatic environments [24]. Thus, external microplastic filters should be regarded as a complementary technology that enhances, rather than replaces, existing wastewater treatment systems.

### 2.3. Emerging Techniques for the Removal of Microplastics from Laundry Wastewater

In addition to conventional treatment processes, membrane-based technologies have proven to be highly effective for the removal of microplastics and microfibers from laundry wastewater [24]. Membrane separation processes, such as MF and UF, can efficiently remove SS, microorganisms, and microplastics; thus, they have been increasingly employed in advanced treatment and water reuse systems [63,64]. For example, MBRs, which integrate activated sludge processes with MF/UF membranes, achieve efficient solid–liquid separation without the need for secondary clarification. As a result, MBR systems can achieve the near-complete removal of SS and substantially reduce microplastic concentrations [53], with a pilot-scale MBR treating municipal wastewater achieving a microplastic removal efficiency exceeding 99%, reducing the concentration from 6.9 particles/L to 0.005 particles/L and outperforming conventional activated sludge processes [21].

A number of studies have investigated the specific use of membrane-based systems for laundry wastewater treatment [47,65,66]. Hybrid processes combining coagulation and UF membranes have demonstrated synergistic effects, achieving a microplastic removal efficiency of up to 96 ± 2% [67]. In addition, high-speed ceramic MF/UF membrane systems have achieved stable and efficient microplastic removal under realistic operating conditions [25]. For example, a comparative analysis of ceramic SiC-based MF and ZrO_2_-based UF membranes has reported removal efficiencies of 98.6% and 99.2%, respectively, with UF membranes producing superior effluent quality due to their finer pore structures [68]. Collectively, these findings highlight the robustness, reliability, and scalability of membrane technologies for microplastic removal and their potential for wastewater reuse.

Nevertheless, despite their high performance, membrane-based systems face several limitations, including high capital and operating costs, membrane fouling, and high energy demands. These constraints have restricted their widespread application, particularly in decentralized or household-scale systems [69,70].

## 3. Development of External Microplastic Filters for Washing Machines

**Figure 1:**
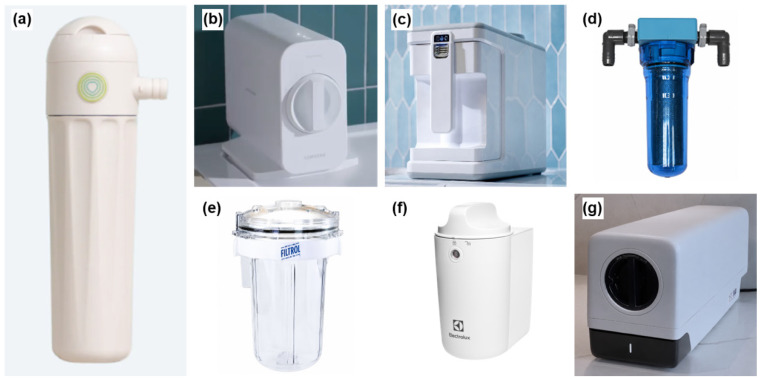
Representative commercial external microplastic filters for washing machines (**a**) PlanetCare 2.0 Microfiber Filter (PlanetCare, Ljubljana, Slovenia), (**b**) Less Microfiber Filter (Samsung Electronics, Suwon, Republic of Korea), (**c**) VORTX filter (CLEANR, Cleveland, OH, USA), (**d**) MicroPlastics LUV-R (Environmental Enhancements, Dartmouth, NS, Canada), (**e**) Filtrol 160 (Wexco, Milaca, MN, USA), (**f**) Microplastic Filter (Electrolux, Stockholm, Sweden), and (**g**) XF3 (Xeros Technology, Rotherham, UK).

In response to growing concerns over microfiber pollution from domestic laundry, governments worldwide are increasingly introducing regulatory and policy measures targeting source control. In particular, the mandatory installation of microplastic filters in washing machines has become a key strategy to reduce emissions at the point of generation (Figure 1). This section reviews recent regulatory developments in major regions, along with corresponding technological responses from industry (Table 2).

France has taken a leading role by enacting the Anti-Waste Law in 2020, which mandates that all new washing machines sold from 2025 must be equipped with microfiber filters. This represents the first legally binding national regulation of its kind and has established a global benchmark for microplastic mitigation policies [71]. In the United States, regulatory efforts are being pursued at the state level. For example, California introduced Assembly Bill (AB) 1628 in 2023 to require filters capable of capturing particles smaller than 100 μm by 2029; although it was vetoed, follow-up legislation (AB 2214) has since been proposed. Similarly, Oregon introduced Senate Bill 526 in 2025, which mandates the installation of microfiber filters in all new washing machines from 2030, along with labeling requirements for proper use and maintenance [72,73].

At the regional level, the European Union has recognized laundry-derived microplastics as an important environmental concern within broader policy frameworks, including Circular Economy Action Plan, although no binding requirements for washing-machine microplastic filters have yet been adopted [74]. In contrast, the United Kingdom has introduced the Microplastic Filters (Washing Machines) Bill, which proposes mandatory installation of washing-machine filters capable of achieving approximately 90% microfiber removal efficiency [75]. Other countries are following similar trajectories; Australia is planning the phased implementation of mandatory filters starting in 2030 under its National Plastics Plan [76], while Ontario in Canada is advancing legislation requiring filters in new machines starting in 2028 [77]. In South Korea, although binding regulations have not yet been enacted, legislative proposals and policy discussions are actively underway, including the proposed Special Act on Microplastics Management and the Ministry of Environment’s Plastic Reduction Roadmap, both of which consider mandatory filter installation as a key measure [78]. Collectively, these developments indicate a clear global trend toward regulatory standardization and the mandatory adoption of microplastic filtration technologies.

Major appliance manufacturers and technology providers are actively responding to these regulatory developments through both operational optimization and product innovation [79]. One widely adopted approach is the implementation of microfiber-reducing washing cycles at the software level. For example, Samsung Electronics, in collaboration with Patagonia, has introduced the “Less Microfiber Cycle”, which reduces fabric friction and water flow, achieving up to a 54% reduction in the release of microfibers [80]. Similarly, LG Electronics has developed a microplastic-reducing cycle accessible via its smart appliance platform [81].

Various filtration technologies have also been commercialized to directly capture microfibers from laundry effluent. These systems can generally be classified as either built-in filters integrated within the washing machines or external filters connected to the drainage line, with the two approaches offering distinct advantages in terms of installation, maintenance, and filtration performance. Reported microfiber removal rates using these technologies typically range from approximately 89% to over 98% depending on the filter configuration, pore size, and operating conditions. However, actual efficiencies can be lower in standardized laundry cycle experiments; an evaluation of three commercial filters reported capture rates of approximately 35–68%, revealing a trade-off between capture efficiency and hydraulic performance [51].

As summarized in Table 3, the pore sizes of commercial external microplastic filters typically range from 45 to 200 µm [82,83]. In general, smaller pore sizes improve microfiber capture efficiency but also accelerate clogging, leading to increased hydraulic resistance and more frequent maintenance. Some commercial designs incorporate bypass channels that divert the flow once the filter becomes clogged, temporarily suspending filtration until the filter is cleaned [84,85]. However, operational performance depends not only on pore size but also on the filter maintenance strategy. For example, passive reusable mesh filters generally require more frequent cleaning than filters equipped with active self-cleaning mechanisms, even when the latter have comparable or finer pore sizes [82,86,87]. Furthermore, direct comparison of reported capture efficiencies remains challenging because testing conditions—including textile type, washing protocol, quantification method, and cycle number—vary considerably among studies and manufacturers. These observations highlight the need for standardized testing protocols to enable meaningful performance comparisons across commercial filtration technologies [51].

In addition to conventional filtration technologies, ceramic membranes are promising high-performance alternatives for the removal of microfibers from laundry wastewater. Evaluation of microfiber transport and retention behavior using real laundry wastewater has demonstrated that polyethersulfone (PES) and zirconia–titania (ZT) membranes can achieve near-complete microfiber retention, with removal efficiencies of 99.88% and 99.96%, respectively, while simultaneously exhibiting enhanced organic matter removal [24]. Ceramic membranes also have an inherently hydrophilic surface that participates in relatively weak interactions with organic foulants, thus reducing their susceptibility to membrane fouling. As a result, ceramic membranes have been reported to exhibit an approximately 1.5–2.0 times lower fouling propensity than conventional polymeric membranes while producing a high-quality permeate, which offers significant advantages for membrane cleaning and water reuse applications [91]. In parallel, innovative filtration concepts incorporating biomimetic structures and reusable filter designs have been actively developed to improve sustainability and long-term operational stability [92,93,94,95]. In addition, complementary consumer-oriented products, including laundry balls and filtration bags, have been introduced as low-cost and easily deployable mitigation strategies for reducing microfiber emissions [23,83].

Overall, there has been a global move toward mandatory microplastic filtration, which has promoted technological innovation and market expansion. However, differences in regulatory standards, such as pore size thresholds, target removal efficiencies, and certification protocols, and practical challenges related to maintenance, user compliance, and long-term performance remain unresolved [96]. There is thus a need for standardized evaluation methods and robust filter designs that improve fouling resistance and durability, ensuring the effective and sustainable implementation of microplastic filtration technologies.

## 4. Upcycling of Captured Microplastics into Membrane Materials

**Figure 2:**
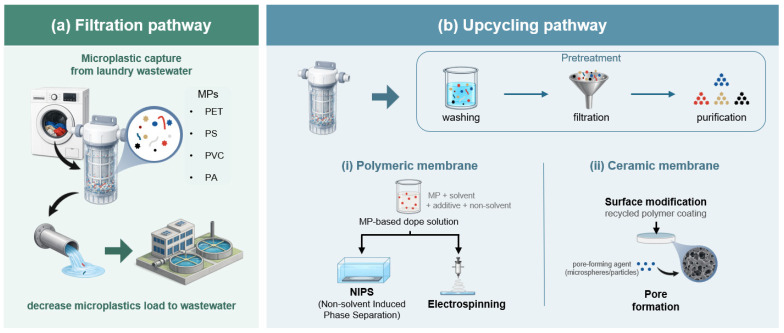
Schematic illustration of the capture and upcycling pathways for laundry-derived microplastics. (**a**) Filtration-based capture of microplastics from laundry wastewater, reducing the release of microplastics into the environment. (**b**) Pretreatment and upcycling of captured microplastics into (i) polymeric membranes through NIPS or electrospinning, and (ii) ceramic membranes through surface modification and pore forming processes.

The microplastics captured from laundry wastewater filters and treatment processes are primarily composed of thermoplastic polymers such as polyethylene terephthalate (PET), polystyrene (PS), and polyvinyl chloride (PVC) [97]. Rather than being disposed of through landfilling or incineration, these materials have the potential to be used as alternative feedstocks for membrane fabrication, in line with circular economy principles [98,99]. This approach simultaneously reduces plastic waste and generates value-added functional materials for water treatment applications (Figure 2).

Captured microplastics are particularly suitable for membrane fabrication because of their thermoplastic nature and favorable physicochemical properties [99,100]. Polymers such as PET, PS, and PVC have glass transition temperatures that are higher than ambient conditions and can be readily processed using solvent dissolution or thermal methods [99,101]. As a result, they are compatible with widely used membrane fabrication techniques, including non-solvent induced phase separation (NIPS) and electrospinning [102]. In addition, a significant fraction of microplastics in laundry wastewater originates from textile fibers, particularly PET and polyamide (PA); these plastics typically have a high molecular weight and good mechanical strength, which are beneficial characteristics for membrane-forming materials [97,103].

The structure and performance of membranes are governed by a four-component system that consists of a polymer matrix, solvent, additives, and non-solvent [104]. While conventional membrane materials such as PVDF, PSf, and PES are widely used [105], waste-derived polymers, including PET, PS, and PVC, are increasingly being investigated as sustainable alternatives. However, the heterogeneous composition and potential impurities present in waste microplastics require the use of pretreatment steps such as washing, filtration, and purification to ensure consistent dope solution properties and membrane performance [99]. A growing body of research has demonstrated the feasibility of fabricating high-performance membranes from waste polymers [99,106,107]. For example, electrospun nanofiber membranes derived from waste PET offer a high porosity (~77%), strong hydrophobicity (contact angle > 130°), and excellent desalination performance in membrane distillation, achieving salt rejection rates of up to 99.9% [108]. NIPS-based membranes incorporating waste PET with additives such as LiCl and zeolite have also been shown to effectively remove heavy metals and turbidity [109], while PEG-modified recycled PET membranes have demonstrated a performance comparable to commercial membranes in MBR systems [110]. Similarly, waste PVC membranes modified with gum arabic exhibit enhanced hydrophilicity, increased permeability, and improved fouling resistance [111], while waste PS-based membranes have proven to be suitable for UF applications, particularly when combined with nanomaterials for surface modification [112].

Captured microplastics can also be employed as functional components in the fabrication of ceramic membranes [113,114]. In particular, PS particles are widely used as pore-forming agents due to their uniform size distribution and clean thermal decomposition behavior [115]. Their incorporation into ceramic matrices such as TiO_2_ and Al_2_O_3_ leads to the formation of well-controlled porous structures with high permeability and tunable mechanical properties [116,117]. In addition, recycled polymer materials have been employed as surface modifiers for ceramic membranes, improving their antifouling performance and energy efficiency [118].

Although previous studies have demonstrated the technical feasibility of producing membranes from waste polymers, translating this approach to microplastics recovered from laundry wastewater remains considerably more challenging. Unlike conventional plastic waste, laundry-derived microplastics are often contaminated with detergent residues, organic matter, and trace metals, necessitating multi-step washing and purification prior to reuse. These additional treatment processes generate secondary wastewater and increase both water consumption and processing costs [119,120].

Another major challenge is the heterogeneous polymer composition of captured microplastics. Laundry-derived microplastics typically consist of mixed polymers, including acrylic, polyamide (PA), polyester (PET), and polypropylene (PP) [3]. Because these polymers are generally incompatible during melt processing, direct recycling without prior separation results in materials with poor mechanical properties, limiting their suitability for membrane fabrication [121]. However, efficient separation of micrometer-sized mixed microplastics remains technically challenging. Existing sorting technologies, such as density separation, Fourier-transform near-infrared (FT-NIR) spectroscopy, and electrostatic separation, were primarily developed for larger and more homogeneous plastic waste streams. Furthermore, density separation alone cannot effectively distinguish polymers with overlapping density ranges, often requiring additional sensor-based sorting or purification steps that increase energy consumption and operational costs [120,122].

Economic feasibility also remains a major limitation. Because laundry-derived microplastics are generally at relatively low concentrations, significant energy and processing are required for their collection, concentration, separation, and purification. Consequently, current recovery technologies are not yet economically competitive with conventional plastic recycling systems [123]. Therefore, although microfiber upcycling represents a promising long-term strategy, further advances in collection, separation, and purification technologies will be essential before its large-scale implementation becomes practical.

Overall, existing laboratory-scale studies, most of which utilize relatively homogeneous conventional plastic waste rather than laundry-derived microplastics, demonstrate the technical potential of producing membrane materials from recycled polymers. However, translating this concept to microplastics captured by external washing-machine filters remains challenging because of the low concentration of recoverable material, heterogeneous polymer composition, contamination by detergent residues and organic matter, and the need for energy-intensive collection, separation, and purification processes. These technical challenges are further compounded by the uncertain economic viability of recovering and processing such small quantities of microplastics.

Consequently, microfiber upcycling should currently be regarded as a promising long-term research direction rather than a readily deployable waste-management strategy. Future studies should evaluate the full environmental and economic performance of this approach through life-cycle assessment, techno-economic analysis, and pilot-scale demonstrations. If these challenges can be overcome, recycling captured microplastics into membrane materials could contribute to a circular economy by transforming waste into value-added materials for water treatment and other environmental applications [124].

## 5. Microbial Presence and Biofouling in Laundry Wastewater

The primary objective of laundering textiles is not only the removal of visible dirt but also the reduction in organic and microbial contamination to hygienically acceptable levels [125]. However, although laundering substantially decreases microbial loads, complete microbial elimination is rarely achieved under typical household washing conditions [126,127]. The degree of microbial reduction is strongly influenced by the operational parameters, including the washing temperature, detergent formulation, and cycle duration. Hygienic decontamination of vegetative pathogens such as *Staphylococcus aureus* and *Pseudomonas aeruginosa* is generally enhanced at higher temperatures, particularly within the 40–60 °C range, and may be improved further with the use of activated-oxygen bleach or longer washing cycles [128,129]. In contrast, low-temperature washing (<40 °C), including cold-water cycles for energy conservation, offers limited microbial inactivation and allows opportunistic and biofilm-forming microorganisms to persist during the laundering process [127,128].

A large fraction of the microorganisms on textiles is detached during the washing cycle due to continuous mechanical agitation and subsequently discharged into the laundry effluent. Laundry wastewater offers a favorable environment for microbial persistence due to the presence of organic substrates derived from body fluids, desquamated skin cells, and textile residues, together with residual surfactants and moderate operating temperatures. Consequently, laundry wastewater is an effective carrier of a diverse range of microbial communities and may support their transport and proliferation under suitable environmental conditions.

**Figure 3:**
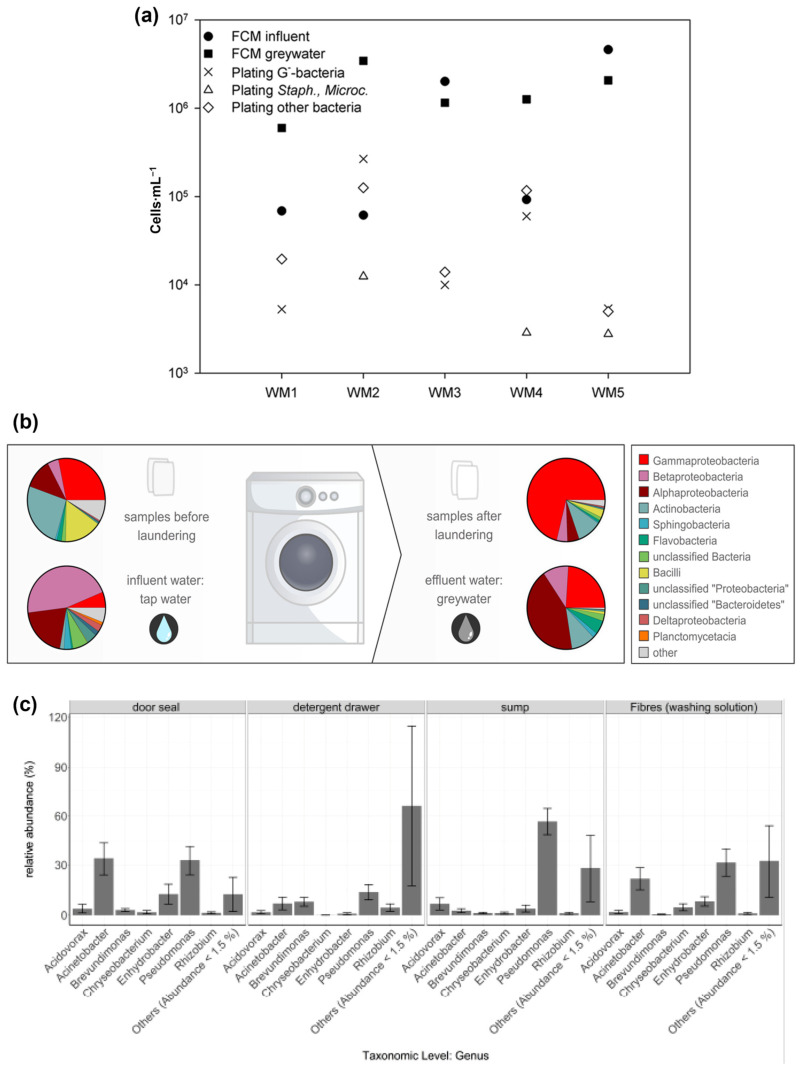
Representative microbial communities and biofouling in laundry-associated wastewater systems: (**a**) bacterial cell counts in influent water and laundry effluent for five household washing machines (WM1–WM5), determined by flow cytometry and selective plating [126], (**b**) relative abundance of bacterial taxa at the class level in influent tap water, effluent water, and cotton textile samples before and after laundering in a household washing machine [126], (**c**) relative abundance of bacterial taxa at the genus level at four sampling sites within household washing machines (door seal, detergent drawer, sump, and fibers recovered from the washing solution) [134].

Microbial characterization studies have reported high levels of microbes in laundry wastewater, with reported bacterial concentrations ranging from approximately 10^4^ to 10^5^ cells/mL based on total cell counts [126]. These authors also found that viable bacterial counts in greywater effluent from washing machines operated at 30 °C were comparable to, or even exceeded, those detected in the influent water, suggesting that laundering may selectively enrich certain detergent-tolerant and biofilm-associated taxa in the discharged wastewater (Figure 3a) [126]. At the community level, laundering significantly alters bacterial composition, with *Gammaproteobacteria* becoming particularly enriched in laundry effluent (Figure 3b). At a finer taxonomic resolution, dominant genera frequently identified in laundry wastewater and washing-machine-associated niches include *Enhydrobacter*, *Staphylococcus*, *Corynebacterium*, and *Pseudomonas* (Figure 3c) [126,130,131,132,133,134]. These microorganisms are characterized by their strong adaptability to fluctuating environmental conditions and resistance to detergents and nutrient-limited environments. In addition to bacteria, fungi and yeasts have also been detected in laundry wastewater, indicating that this effluent supports diverse microbial communities [127,135,136].

Several studies have reported the presence of clinically relevant microorganisms on laundered textiles and within internal components of household washing machines, including ESKAPE (*Enterococcus faecium*, *Staphylococcus aureus*, *Klebsiella pneumoniae*, *Acinetobacter baumannii*, *Pseudomonas aeruginosa*, and *Enterobacter* spp.) pathogens, which are recognized for their ability to acquire and disseminate antimicrobial resistance. Whitehead et al. reported that public washing machines can mediate microbial cross-contamination between laundered textiles, with multiple ESKAPE pathogens, including *S. aureus*, *K. pneumoniae*, and *A. baumannii*, detected on the laundered fabrics [137]. Although their study did not directly analyze laundry effluent, their findings supported concerns regarding the redistribution of clinically important microorganisms during laundering processes. In a notable hospital-associated case, Schmithausen et al. [138]) reported persistent colonization of an extended-spectrum β-lactamase (ESBL)-producing *Klebsiella oxytoca* within the residual water and detergent-drawer compartments of a household washing machine used in a German perinatal care setting. The organism was subsequently transmitted to newborns via contaminated laundered textiles until the washing machine was removed from service [138].

A comparison of previous studies investigating microbial communities associated with washing machines is summarized in Table 4. Reported microbial loads vary considerably among sampling sites and are consistently highest at persistently wet, difficult-to-clean locations, such as front-load door seals (up to ~6.50 log CFU/swab) and detergent-drawer water port (up to ~6.28 log CFU/swab), while lower loads are generally observed on drum walls and agitator surfaces. Across independent studies employing different sampling and analytical methods, *Pseudomonas*, *Acinetobacter*, *Staphylococcus*, and *Micrococcus* were consistently identified as dominant bacterial genera, together with fungi such as *Rhodotorula*, *Candida*, and *Cladosporium*. Importantly, all of these observations were obtained from internal washing-machine components or washing-related water; no studies have directly characterized microbial communities on external microplastic filters. Nevertheless, the consistently high microbial loads observed at wet, particle-retaining internal sites suggest that household washing machines provide conditions favorable for microbial colonization. Whether comparable microbial accumulation and biofilm formation occur on external microplastic filters remains unknown and warrants dedicated investigation.

The coexistence of organic matter, surfactants, suspended solids, and microplastic fibers in laundry wastewater also promotes microbial aggregation and biofilm formation. Microorganisms can readily associate with particulate matter, including synthetic microfibers, forming complex aggregates that promote microbial persistence and transport. These fiber-associated microbial assemblages, commonly referred to as the plastisphere, can become biofilm-producing communities that develop on hydrophobic polymeric surfaces [143,144]. Due to their persistent and hydrophobic nature, microplastic surfaces provide favorable conditions for rapid microbial adhesion, irreversible attachment, and subsequent EPS-mediated biofilm development [143,145]. Through repeated washing cycles, these microbial communities are continuously discharged into wastewater streams, contributing to elevated microbial loads and increasing the potential for downstream contamination.

These studies demonstrate that clinically relevant microorganisms can colonize laundered textiles and internal washing-machine components. However, whether similar microbial colonization occurs on external microplastic filters has not yet been directly investigated. Importantly, external microplastic filters installed in washing machine drainage systems are the primary interception point for both microplastic fibers and associated microorganisms. The accumulation of organic matter and retained particulates within these filters creates favorable conditions for microbial attachment and proliferation, potentially transforming the filters into hotspots for biofilm formation. Biofilm formation has been well documented on the internal wetted components of household washing machines. For example, Gattlen et al. identified pronounced biofilm growth on built-in drain filters, outer-drum metal surfaces, and rubber tubing, with approximately 30% of the recovered isolates classified as potential human pathogens [142]. Although external microplastic filters similarly retain microfibers and organic matter between cleaning intervals, no studies have directly characterized microbial colonization or biofilm formation on these filters. Therefore, the potential for biofilm accumulation and associated hygiene risks on external filters is inferred from analogous systems and should be regarded as an important research gap requiring experimental validation. These observations highlight the importance of considering microbial dynamics and biofouling behavior in the design and operation of laundry wastewater filtration systems, with microbial accumulation on filter surfaces potentially affecting filtration efficiency, hydraulic resistance, pressure buildup, and long-term operational stability.

## 6. Microbial Colonization of Microplastic Filters

**Figure 4:**
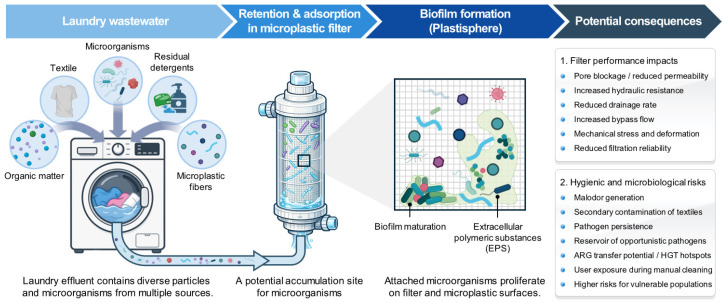
Schematic illustration of microbial accumulation and biofilm development on microplastic filters in washing machines.

As discussed in the previous section, laundry wastewater contains a diverse range of microbial communities that are continuously transported during the washing process. These microorganisms, originating from textiles, human skin, and environmental sources, are discharged with the laundry effluent and often pass through washing machine-mounted microplastic filters. During this process, suspended microorganisms are likely to be physically retained or adsorbed onto the filter surface along with microplastic fibers and other particulates [146]. Accordingly, microplastic filters can become potential accumulation sites where microorganisms can concentrate and persist (Figure 4). Despite this, studies directly investigating microbial communities or biofilm formation within washing machine microplastic filters remain scarce, highlighting a critical knowledge gap in understanding their microbiological behavior.

The structure and function of microplastic filters enhance their susceptibility to microbial colonization. These filters typically consist of fine mesh or porous media designed to capture small particles, resulting in a high surface area available for microbial attachment [147]. In addition, microplastics retained within the filter can act as a substrate for microbial adhesion. Due to its hydrophobic properties, the microplastic surface readily facilitates microbial attachment and supports the formation of the plastisphere [144,148]. These plastisphere communities can include opportunistic or pathogenic microorganisms and exhibit unique functional characteristics, thus increasing the potential hygienic risks [149,150].

Microplastic filters also accumulate the organic matter and residual detergents present in laundry wastewater. These substances can act as carbon and nutrient sources, promoting microbial growth within the filter matrix [37,147]. Biofilm development generally proceeds through sequential stages: reversible attachment, irreversible attachment, EPS-driven maturation, and partial detachment followed by recolonization [151]. During maturation, attached microorganisms secrete EPS, which strengthen surface adhesion, bind retained microplastic fibers, organic matter, and microbial cells into a cohesive matrix, and protect embedded microorganisms from environmental stresses [152]. This process facilitates the formation of complex biofouling layers composed of microorganisms, microplastics, and accumulated organic matter [153]. Over time, these biofilms can become increasingly dense and resistant to removal via physical cleaning.

The surfactants present in laundry wastewater further complicate this colonization process. Synthetic surfactants such as sodium dodecyl sulfate (SDS) are amphiphilic compounds that reduce surface tension and can inhibit microbial attachment, and they have been reported to suppress biofilm formation in a variety of industrial, medical, and environmental systems [154]. Inhibitory effects have been observed above threshold concentrations [155]. However, the applicability of these findings to external washing-machine filters remains uncertain. Most available studies have been conducted using single microbial strains and SDS-based model systems, whereas the surfactants most commonly found in laundry detergents, such as linear alkylbenzene sulfonate, have received comparatively little attention. In addition, surfactant effects are highly substrate-dependent, and biofilm inhibition observed on some materials may not apply to hydrophobic plastic surfaces, where microbial attachment can instead be promoted [155]. Furthermore, because surfactant antimicrobial activity depends on both concentration and exposure time, the relatively short duration of household washing cycles may be insufficient to eliminate mature biofilms or microorganisms embedded within retained microfiber layers [136]. Consequently, the overall influence of laundry surfactants on biofilm development in external microplastic filters remains unresolved and requires further investigation.

The development of microbial biofilms within external microplastic filters may have important implications for both filtration performance and hygiene. Under realistic washing-machine operating conditions, the accumulation of retained microfibers on commercial filters has been shown to increase the pressure gradient across the filter and reduce drainage flow over repeated wash cycles, illustrating the trade-off between microfiber capture efficiency and hydraulic performance [51]. Similarly, progressive clogging and flow-rate reduction have been identified as key factors limiting the long-term performance of external filters [96]. Biofilm growth is expected to further exacerbate this physical fouling by colonizing the retained microfiber layer, narrowing the effective pore size, and increasing hydraulic resistance [156,157]. Consequently, excessive fouling may reduce drainage rates, increase mechanical stress on the filter, and potentially induce bypass flow, thereby compromising filtration performance and reliability [158].

However, these mechanisms are inferred primarily from studies of MBRs and granular filtration systems rather than from external washing-machine microplastic filters. To date, no studies have quantitatively measured the contribution of biofilm development to pressure-drop increases over successive washing cycles. Therefore, systematic, cycle-resolved measurements that distinguish physical clogging by retained microfibers from biofilm-induced hydraulic resistance remain an important research priority.

In addition to operational issues, microbial growth within filters may pose hygienic concerns. The proliferation of odor-causing bacteria, such as *Moraxella osloensis*, which has been identified as a major contributor to washing machine malodor, can lead to unpleasant odors and lower user acceptability [134,141,159,160]. In addition, if filters are not properly maintained, microbial metabolites and volatile compounds may be released back into the washing system or indoor environment, potentially resulting in the secondary contamination of laundered textiles [126,127].

The potential hygiene risks associated with external microplastic filters remain largely hypothetical, as no studies have directly characterized microbial colonization or pathogen persistence on these filters. Nevertheless, washing machines are recognized as potential reservoirs and transmission routes for opportunistic microorganisms, particularly when laundering contaminated textiles. For example, laundry associated with healthcare-related activities, such as workwear worn by healthcare professionals or textiles used in home-based patient care, may introduce clinically relevant microorganisms into household laundry wastewater [161]. By analogy with other particle-retaining filtration systems, such microorganisms could accumulate within external filters and persist through repeated washing cycles, potentially increasing the risk of household-level recontamination. Likewise, biofilms are well known to facilitate horizontal gene transfer, including the dissemination of antimicrobial resistance genes [162]. If comparable biofilms develop on external microplastic filters, they could similarly contribute to the persistence and spread of antimicrobial resistance in household environments. In addition, because most commercial external filters require manual disassembly, cleaning, and disposal of retained debris, maintenance activities could expose users to accumulated microorganisms and biofilm-associated contaminants. However, these potential hygiene risks are inferred from studies of internal washing-machine components and related filtration systems rather than from direct observations of external microplastic filters. Experimental studies are therefore needed to evaluate microbial colonization, biofilm formation, and their implications for household hygiene.

## 7. Conclusions

Laundry-derived microplastic fibers are a major source of environmental microplastic pollution due to their continuous release during domestic laundering and persistence in aquatic environments. Washing machine-mounted microplastic filters are a promising source-control technology capable of reducing microfiber discharge before wastewater release. This review summarized recent advances in microfiber capture technologies, including internal and external filtration systems and membrane-based approaches to laundry wastewater treatment. In addition to microfiber removal, the potential upcycling of captured microplastics into polymeric and ceramic membrane materials is a sustainable strategy for waste valorization and circular resource utilization. Membrane-based systems, particularly ceramic membranes, have demonstrated high microfiber removal efficiencies and favorable antifouling characteristics, highlighting their potential for advanced water treatment applications. Importantly, this review also emphasized the overlooked issue of microbial accumulation and biofouling within laundry wastewater filtration systems. Laundry effluent contains various microorganisms that can become associated with retained microplastic fibers and accumulated organic matter, promoting biofilm formation within the filter. This biofouling may negatively affect filtration performance, operational stability, and household hygiene, while also raising concerns regarding pathogen persistence and antibiotic resistance dissemination.

## Figures and Tables

**Table 2:** Summary of legislative and policy measures for mandatory microplastic filters in washing machines by country/region.

County/Region	Year Enacted/Proposed	Key Legislative or Policy	Implementation Timeline	Required Pore Size/Removal Efficiency	Ref.
France	2020	Mandatory filter in all new washing machines	From 2025	Not specified	[71]
California (USA)	2023	Mandatory filters ≤ 100 μm	Target 2029	≤100 μm	[72]
Oregon (USA)	2025	Mandatory filters ≤ 100 μm in new washing machines	From 1 January 2030	≤100 μm	[73]
European Union	Proposed	Mandatory filters with > 90% removal efficiency in new machines	TBD	Not specified	[74]
United Kingdom	2024	Bill mandating filters in all new washing machines	Second reading in 2025	>90% removal	[75]
Australia	2021	Phased introduction of filters in residential and commercial machines	From 1 July 2030	Not specified	[76]
Ontario (Canada)	Proposed	Filters mandatory in all new machines	From 2028	≤100 μm	[77]
South Korea	Proposed	Special Act includes consideration of mandatory filters	TBD	Not specified	[78]

**Table 3:** Specifications and reported performance of representative commercial external microplastic filters for washing machines shown in Figure 1.

Product	Pore Size	Capture Efficiency	Clogging Behavior	Maintenance Interval	Ref.
(a) PlanetCare 2.0	–	98%	Auto-stop with error alert on drain-path obstruction	~20 washes/cartridge; cartridges returned to manufacturer	[88]
	200 µm	25 ± 20%	Multi-layer medium distributes capture through depth, limiting clogging		[83]
(b) Less Microfiber Filter	65–70 µm	≤98%	Blade-sweep suppresses mesh accumulation	Clean ~every 30 cycles	[89]
(c) VORTX	50 µm	>90%	Vortex + self-cleaning limit mesh accumulation	Pod replaced ~every 5 washes; mesh cleaned/reused ~every 20–30	[87]
(d) MicroPlastics LUV-R	150 µm	87–100%	Dynamic capture (lint raises retention but obstructs housing); back-pressure <5 psi after ~1 week	Clean ~every 2–3 cycles	[86]
	285 and 175 µm	29 ± 15%		Clean ~every 3 week (4-person household)	[83]
	150 µm	87%		-	[23]
(e) Filtrol 160	100 µm	89–100%	Bypass opens when saturated (prevents overflow, suspends capture)	Clean ~every 8–10 cycles	[85]
		≤87%			[84]
(f) Microplastic Filter	45 µm	≤90%	Red float indicates obstruction	Clean weekly; ~6-month service life	[82]
(g) XF3	60 µm	>99%	Self-cleaning spin minimizes emptying	Lasts washing-machine lifetime	[90]
		78%	–	Captured fibers to household waste ~every 30 cycles	[83]

For products (a), (d), and (g), the reported values include data from both the current and previous product generations.

**Table 4:** Summary of previous studies investigating microbial communities in household washing machines and washing-related water.

Ref.	Analysis Methods	Sampling Sites		Microbial Counts	Dominant Taxa
[127]	Culture-based method; 16S rRNA gene/ITS amplicon sequencing	Top-load machines	Drum wall	Bacteria: 3.71 ± 1.02 log CFU/swab Fungi: 1.53 ± 0.34 log CFU/swab	Bacteria: *Pseudomonas*, *Micrococcus*, *Sphingomonas*, *Staphylococcus* Fungi: *Aspergillus*, *Cladosporium*, *Penicillium*, *Trichoderma*
			Drum gasket	Bacteria: 3.79 ± 1.73 log CFU/swab Fungi: 2.27 ± 0.87 log CFU/swab	
			Drum lower edge	Bacteria: 2.05 ± 0.9 log CFU/swab Fungi: 1.27 ± 0.07 log CFU/swab	
			Agitator spindle	Bacteria: 2.68 ± 0.36 log CFU/swab Fungi: 1.1 ± 0.5 log CFU/swab	
			Fabric softener reservoir	Bacteria: 2.46 ± 1.49 log CFU/swab Fungi: 1.30 ± 0 log CFU/swab	
		Front-load machines	Drum wall	Bacteria: 3.32 ± 1.44 log CFU/swab Fungi: 1.68 ± 0.53 log CFU/swab	
			Door seal	Bacteria: 6.50 ± 2.46 log CFU/swab Fungi: 3.25 ± 1.4 log CFU/swab	
			Water spray port	Bacteria: 4.90 ± 2.94 log CFU/swab Fungi: 2.48 ± 1.64 log CFU/swab	
			Drum lower edge	Bacteria: 5.02 ± 2.67 log CFU/swab Fungi: 2.39 ± 1.11 log CFU/swab	
			Detergent drawer	Bacteria: 4.71 ± 2.80 log CFU/swab Fungi: 2.87 ± 1.63 log CFU/swab	
			Detergent drawer water port	Bacteria: 6.28 ± 2.54 log CFU/swab Fungi: 3.63 ± 1.35 log CFU/swab	
[136]	Culture-based method; 16S rRNA gene amplicon sequencing	Washing solution elution		Bacteria: >700 CFU/100 cm^2^ Fungi: >50 CFU/100 cm^2^	Bacteria: *Pseudomonas*, *Enhydrobacter*, *Brevibacterium*, *Acinetobacter* Fungi: *Cyphellophora*
		Outer wall, base, rubber ring		-	*Enhydrobacter*, *Brevibacterium*
[139]	Culture-based method; MALDI Biotyper	Detergent drawer		Bacteria: 1.1 ± 0.74 × 10^4^ CFU/cm^2^	*Rhizobium radiobacter*, *Pseudomonas*, *Acinetobacter*, *Staphylococcus*, *Micrococcus*
		Detergent drawer chamber		Bacteria: 4.2 ± 3.0 × 10^4^ CFU/cm^2^	*Pseudomonas*, *Acinetobacter*, *Staphylococcus*, *Micrococcus*
		Top of rubber door seal		Bacteria: 11.1 ± 9.2 × 10^1^ CFU/cm^2^	*Micrococcus*, *Staphylococcus*, *Acinetobacter*, *Pseudomonas*
		Bottom of rubber door seal		Bacteria: 3.1 ± 1.9 × 10^4^ CFU/cm^2^	*Micrococcus*, *Acinetobacter*, *Pseudomonas*, *Staphylococcus*
[134]	16S rRNA gene pyrosequencing	Detergent drawer		-	*Pseudomonas*, *Brevundimonas*
		Rubber door seal			*Acinetobacter*, *Pseudomonas*, *Enhydrobacter*, *Moraxella osloensis*
		Sump			*Pseudomonas*, *Acidovorax*
		Fibers collected from the washing solution			*Pseudomonas*, *Acinetobacter*, *Enhydrobacter*, *Moraxella osloensis*
[135]	Culture-based method	Detergent drawer, Rubber door seal		-	Bacteria: *Micrococcus luteus*, *Pseudomonas aeruginosa*, *Sphingomonas* Fungi: *Fusarium oxysporum*, *Fusarium solani*, *Candida parapsilosis*, *Exophiala phaeomuriformis*
[126]	Culture-based method; 16S rRNA gene pyrosequencing	Effluent Water		Bacteria: 1.32 × 10^4^~4.06 × 10^5^ cells/mL	*Enhydrobacter*, *Staphylococcus*, *Corynebacterium*, *Pseudomonas*
[140]	16S rRNA gene/ITS pyrosequencing	Rubber door seal		-	Bacteria: *Acinetobacter*, *Brevundimonas*, *Pseudomonas* Fungi: *Meyerozyma*/*Candida, Cladosporium*, *Nectria*, *Wallemia muriae*, *Rhodotorula mucilaginosa*
		Detergent drawer			Bacteria: Proteobacteria (p), Rhizobiales (o), *Ochrobactrum* Fungi: *Candida parapsilosis*, *Rhodotorula minuta*, *Rhodotorula slooffiae*
[141]	Culture-based method	Rubber door seal		Bacteria: ~10^3^~10^4^ CFU/cm^2^	-
		Detergent drawer		Bacteria: ~10^−1^~10^3^ CFU/cm^2^	
[142]	Culture-based method	Rubber door seal		-	*Pseudomonas putida*, *Pseudomonas aeruginosa*, *Klebsiella pneumoniae*

## Data Availability

No new data were created or analyzed in this study. Data sharing is not applicable to this article.
